# Efficacy of a reduced-dosage PRRS MLV vaccine against a NADC34-like strain of porcine reproductive and respiratory syndrome virus

**DOI:** 10.3389/fvets.2024.1493384

**Published:** 2025-01-06

**Authors:** Xinyu Yan, Jiayu Liu, Fengxiong Yue, Yan Lin, Yan Li, Wensi Wu, Shan Zhao, Xiaobo Huang, Qin Zhao, Yiping Wen, Sanjie Cao, Senyan Du, Nanfang Zeng, Qigui Yan

**Affiliations:** ^1^College of Veterinary Medicine, Sichuan Agricultural University, Chengdu, China; ^2^Giantstar Farming and Husbandry Co., Ltd., Chengdu, China; ^3^Chengdu SG-Biotech Co., Ltd., Chengdu, China

**Keywords:** MLV vaccine, NADC34-like PRRSV, vaccine, efficacy, different dosages

## Abstract

**Introduction:**

After being discovered for the first time in China in 2017, porcine reproductive and respiratory syndrome virus (PRRSV) NADC34-like strains have become the prevalent strain of PRRSV in certain regions of China. Our previous study showed that reduced Ingelvac PRRS MLV vaccination dosages against NADC30-like *CF* PRRSV had a better protection effect than the normal dosage. However, the protective effect of reduced dosages vaccination of Ingelvac PRRS MLV against NADC34-like PRRSV is unclear. Therefore, this study compared the effectiveness of 0.1 and 1 dosages against a NADC34-like PRRSV infection using commercial PRRSV vaccines, Ingelvac PRRS MLV, which have been widely utilized in China.

**Methods:**

In this study, we immunized piglets with two different dosages of the MLV vaccine and infected piglets within a nasal way with NADC34-like CF PRRSV at 42 days post-vaccination. We observed the changes in growth performance before and after the NADC34-like PRRSV DX strain challenge and the protective effect of different vaccine dosages through multiple assays.

**Results:**

After the challenge, the piglets from the challenge control group displayed clinical signs typical of PRRSV infection, including transient fever, high viremia, mild clinical symptoms, and histopathological changes in the lungs and lymph nodes, which indicates DX is a virulent virus. Without the challenge, the average daily gain of the non-immunized group at 5 weeks after the vaccination is greater than that of the 0.01 dosage group than that of the 1 dosage group, which proved that the commercial MLV vaccine has a negative effect on the growth performance of pigs and this effect may be dose-dependent. After the NADC34-like PRRSV challenge, there was no difference in average daily gain between the immunized pigs and pigs from the challenge control group. From the perspective of clinical score, gross lung lesions, and microscopic lesions, immunization with MLV vaccine can indeed relieve symptoms and lesions caused by the virus, and 0.1 dosage vaccination has a better effect in these aspects. Also, both dosages of MLV immunization shortened viremia with similar effects.

**Discussion:**

Our research suggests that the MLV vaccine can provide piglets with some protection against NADC34-like PRRSV and the 0.1 dosage Ingelvac PRRS MLV vaccination showed greater benefits in our study. Therefore, considering the cost, side effects, and subsequent protective effects, we can adjust the immune dosage appropriately after further investigation to ensure safety, improve production efficiency, and reduce immunization costs.

## Introduction

Porcine reproductive and respiratory syndrome (PRRS) is an acute infectious disease caused by the porcine reproductive and respiratory syndrome virus (PRRSV) (1, 2). The virus was an enveloped RNA virus with a genome length of approximately 15.3 kb and was first discovered in North Carolina, United States in 1987 (3, 4). For over 30 years, this disease has been occurring continuously and has currently become one of the major diseases affecting the healthy development of the global swine industry (5–7). Both vertical and horizontal transmission of the virus can result in reproductive problems such as stillbirth and abortion in pregnant sows, piglet mortality, and stunted growth and development (8).

PRRSV belongs to the genus Betaarterivirus, family Arteriviridae, and order Nidovirales. Based on the global PRRSV classification system, usually, PRRSV has two species: Betaarterivirus suid 1 (PRRSV-1, represented by Lelystad Virus strains) and Betaarterivirus suid 2 (PRRSV-2, represented by VR-2332 strains) (9). The main prevalent strain in China is PRRSV-2 (3, 10). PRRSV-2 strains 1–7-4 (IA/2014/NADC34, IA/2013/ISU1, and IN/2014/ISU-5) were first discovered in North Carolina and Iowa, United States in 2013 (11). Later in 2017, Zhang et al. reported that LNWK96 and LNWK130, which had a 100-amino-acid deletion in the Nsp2 region isolated from Liaoning pig farms in 2017, may have originated from the 1-7-4 strain prevalent in the United States, providing the first evidence of the emergence of NADC34-like PRRSV in China (12). The NADC34-like strain’s distribution started to progressively widen in 2019 (13–15). At present, NADC34-like PRRSV has become the main prevalent strain in some regions of China (10, 16–18).

At present, the most effective method to prevent PRRS is still vaccine immunization (19). In China, the most commonly used types of vaccine are modified-live-virus (MLV) vaccines (CH-1R, JXA1-R, VR-2332, etc.) (20). The live-attenuated PRRS vaccine can promote humoral immunity and cellular immunity (21, 22). Compared to the inactivated vaccine, it has a longer immune duration and a superior immune response (22). The common consensus is that vaccinations of PRRSV MLV against heterologous strains can help relieve symptoms (23–25). A study has suggested that current vaccinations may provide partial protection against PRRSV strains of linage 1 (NADC34-like, sublineage 1.5) (26).

According to our understanding, the industry is trying to reduce the cost of vaccines by lowering vaccination dosages. Additionally, in our previous research, piglets vaccinated with reduced dosages of Ingelvac PRRS MLV can provide better protection for the NADC30-like PRRSV (sublineage 1.8) challenge. Whether a reduced dosage of MLV vaccine can provide protection against NADC34-like strains and influence production performance remains to be investigated. We hope to comprehensively analyze and evaluate the cost and benefit of vaccine use from the perspective of its impact on growth performance. In this study, we investigate both the pathologic and productive effects of the 0.1 dosage and 1 dosage of this commercially available live-attenuated vaccine by animal experiments.

## Materials and methods

### Virus and MLV vaccine

Ingelvac PRRS MLV vaccine, purchased from Boehringer-Ingelheim, Germany, is one commercial live-modified PRRSV vaccine that is derived from the virus VR-2332 strain. The viral used in this work was DX (PQ217625), an isolated NADC34-like PRRSV provided by Chengdu SG-Biotech Co., Ltd.

### Animal trials for vaccination and challenge

A total number of thirty-six 3-week-old piglets that were free of the porcine circovirus 2 (PCV2), classical swine fever virus (CSFV), pseudorabies virus (PRV), and PRRSV were randomly assigned to six groups, each consisting of six piglets. The Ingelvac PRRS MLV vaccine was diluted to 1 dosage (10^4.8^ TCID50) and 0.1 dosage (10^3.8^ TCID50). Piglets were intramuscularly (IM) inoculated with phosphate-buffered saline (PBS, including two groups), 1 dosage (including two groups), and 0.1 dosage of Ingelvac PRRS MLV vaccine (including two groups). Forty-two days after the first vaccination (42-day post-vaccination, 42 dpv), the piglets were intranasally challenged with the NADC34-like DX virus (2 mL total, 10^5.5^ TCID50/ml, including three groups with different immune conditions), and the piglets from the other three negative control groups were treated with an equal amount of cell culture. The specific group information is shown in Table 1. All piglets were subject to free feeding during the experiment.

**Table 1:** Information of groups.

Time node	A	B	C	D	Challenge control	Mock control
Vaccination	0.1 dosage	0.1 dosage	1 dosage	1 dosage	−	−
Challenge	+	−	+	−	+	−

The rectal body temperatures were collected for 2 weeks after the vaccination. After the challenge, the rectal body temperatures and clinical signs of the piglets were recorded every day. During the experiment, the status of the piglets was recorded by scoring, including gross clinical scores (GCSs), respiratory clinical scores (RCSs), and nervous signs scores (NSSs). The specific scoring rules are shown in Table 2. All piglets were humanly euthanized at 21 dpc (days post-challenge). Body weight and total feed intake were measured every week throughout the experiment.

**Table 2:** Clinical sign scoring system used for infected pigs.

	Symptom types	Evaluation criterion	Score
Gross clinical scores, GCS	Temperature	T ≤ 39.9°C	0
		40.0°C ≤ T ≤ 40.9°C	1
		41.0°C ≤ T	2
	Appetite	Normal	0
		Loss of appetite	1
	Mentality	Normal	0
		Unclear consciousness/drowsiness	1
	Skin	Normal	0
		Cyanochroia	1
Respiratory clinical scores, RCS	Respiratory disease	Normal	0
		Rapid breathing during tension	1
		Rapid breathing during rest	2
		Rapid breathing and difficulty breathing during rest	3
		Severe shortness of breath, difficulty breathing, irregular breathing, and difficulty breathing	4
	Cough	Normal	0
		Cough	1
	Runny nose	Normal	0
		Runny nose	1
Nervous signs scores, NSS	Neurological symptoms	Normal	0
		Tremble	1
		Ataxia	2
		Arm pull	3
		Paralysis	4

*Usual condition: total scores = GCS + RCS + NSS; If piglet died: total scores GCS + RCS + NSS + 5; 0 ≤ total scores ≤ 20.

### Serology and viremia test

The blood samples of piglets were drawn after immunization as well as at 21, 42 days post-vaccination, and 7, 14, and 21 days post-challenge to identify specific antibodies to PRRSV. The IDEXX PRRS 2XR Porcine Reproductive and Respiratory Syndrome Virus Antibody Test Kit (IDEXX Laboratories) was used as directed by the manufacturer to assess PRRSV-specific ELISA antibody titers. S/P ratios were used to report PRRSV-specific antibody titers, and the serum samples were deemed positive if the S/P ratio was 0.4 or greater.

Total RNA was extracted from the serum samples using the Virus DNA/RNA Extraction Kit 2.0 (Vazyme). After reverse transcription using the HiScript II Q RT SuperMix for qPCR (Vazyme), real-time PCR was conducted to detect the cDNA from samples from 1, 3, 5, 7, 10, 14, 21 days post-challenge using the AceQ qPCR Probe Master Mix (Vazyme). The qPCR primers and probe were designed according to the conserved sequence of the M gene by Chengdu SG-Biotech Co., Ltd. The primers of real-time PCR were PRRSV MF1: 5′-TCCAGATGCCGKTTGTGCTT-3′; MF2: 5′-TCCAGATRCCGGTTGTGCTT-3′; MF3: 5′-TCTAGATGCCGTTTGTGCYT-3′; PRRSV MR: 5′-ACGACAAATGCGTGGTTATCA-3′. The TaqMan probe was synthesized as 5′-FAM-CCCTGCCCACCACGT-MGB-3′. The conditions for amplification were 38°C for 2 min and 95°C for 5 min, then 40 cycles of 95°C for 10 s and 60°C for 30 s. The viral load of each sample was calculated by the equation (y = 40.18–3.25x) constructed previously by Chengdu SG-Biotech Co., Ltd.

### Histopathology and immunohistochemistry examination

**Figure 1:**
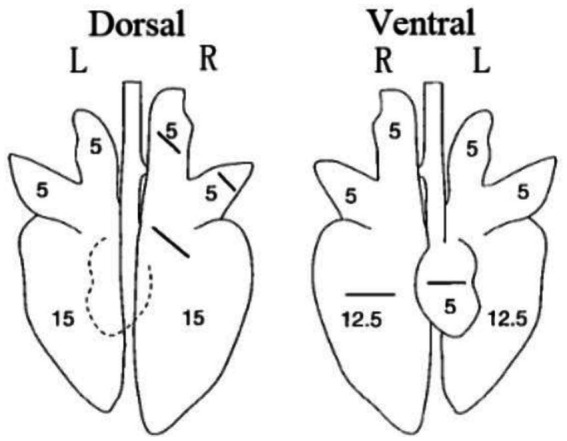
Scoring criteria for gross lesions in pulmonary autopsy.

All piglets were humanly euthanized at 21 dpc. At necropsy, the lungs were observed and the lesions were recorded. The three parts of the lung were fixed in 10% buffered neutral formalin for hematoxylin and eosin (H&E) and immunohistochemistry staining. Photos taken with a 200× microscope were used to visualize the slides. The lungs were observed and the lesions were recorded and scored according to Figure 1 and Table 3.

**Table 3:** Scoring criteria for lung tissue section.

Score	Pathological condition
0	No obvious lesions
1	Slight pathological changes, thickening of the alveolar wall or the infiltration of inflammatory cells or stasis or slight shedding of mucosal epithelial cells
2	Interstitial pneumonia and slight focal distribution
3	Interstitial pneumonia, moderate diffuse distribution, or severe focal distribution, more than 2/5 of the lesion area
4	Interstitial pneumonia, severe diffuse distribution, and pathological tissue area accounted for more than 4/5

### Statistical analysis

All data were expressed as the mean value of 5 piglets ± SEM. Using the GraphPad Prism 9 program (San Diego, CA), statistical analyses were carried out by performing a two-way ANOVA and then Tukey’s *t*-test. When *p* < 0.05, differences were deemed statistically significant.

## Results

### Clinical presentation and piglet growth performance

**Figure 2:**
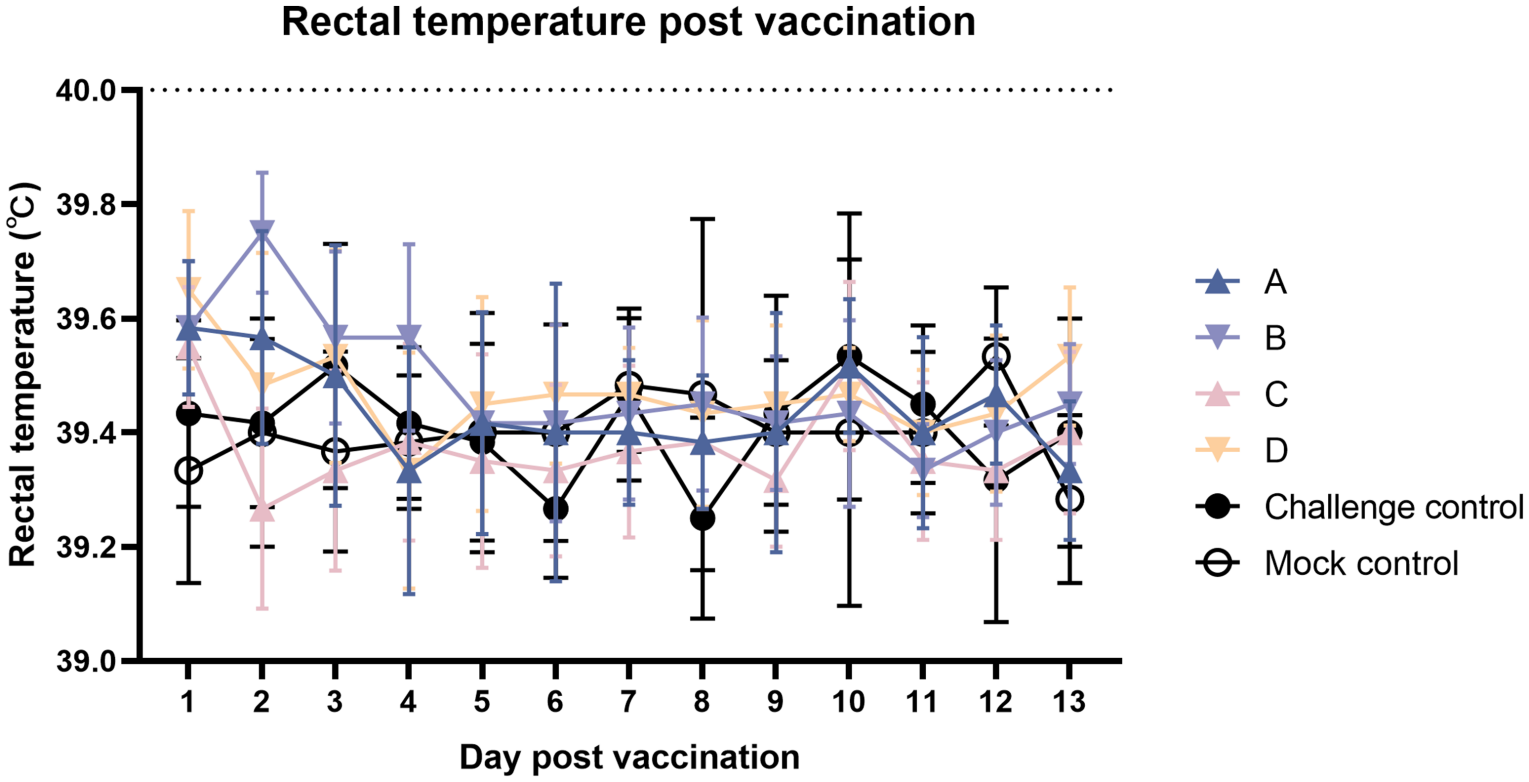
Comparison of mean rectal temperatures (±S.D.) after the vaccination. Clinical fever was set at 40°C.

As shown in Figure 2, after immunization, the body temperature was measured continuously for 2 weeks, and the body temperature of the immune group fluctuated but did not exceed 40°C.

**Figure 3:**
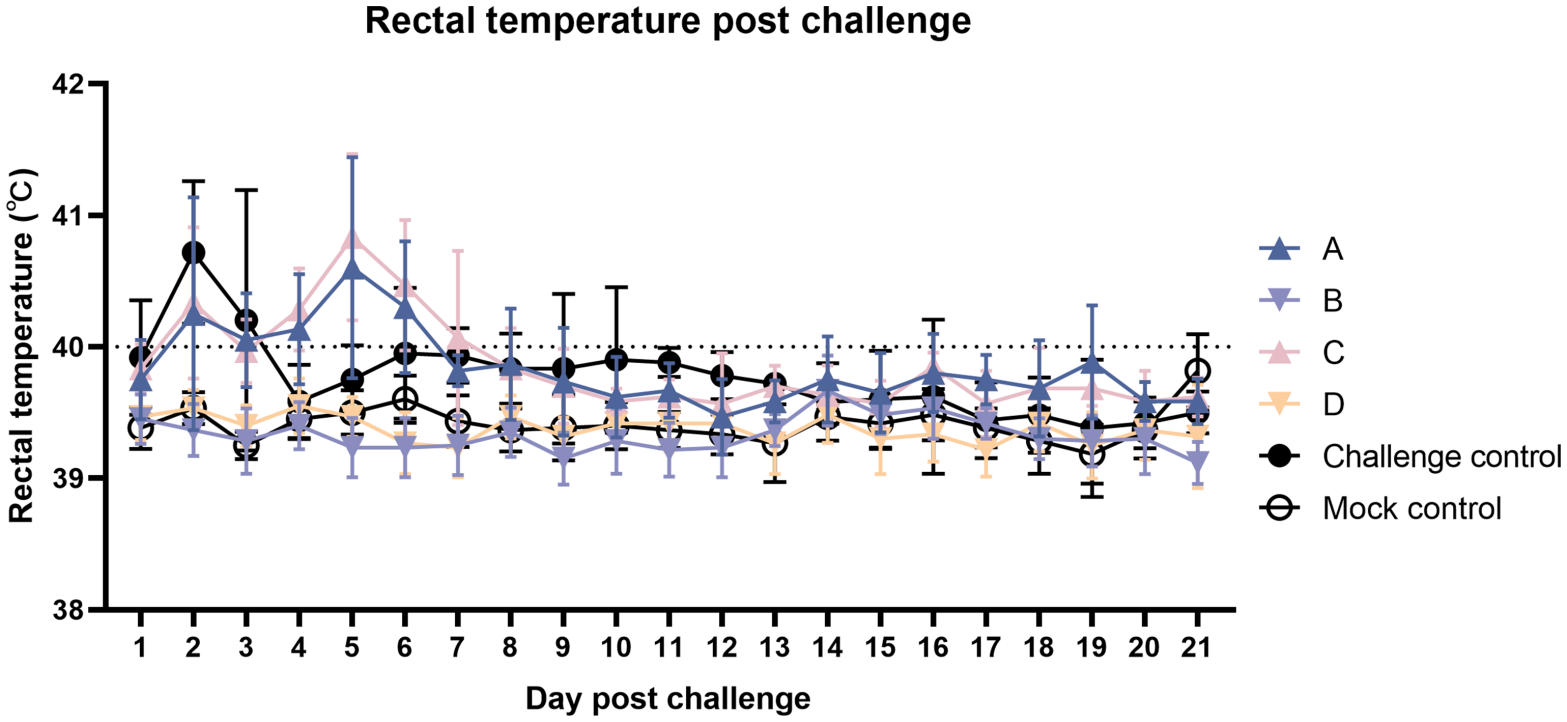
Comparison of mean rectal temperatures (±S.D.) after the challenge. Clinical fever was set at 40°C.

According to Figure 3, after the challenge, each challenge group had different degrees of fever. The fever in the challenge group was the most critical in the first 1–3 days, followed by sporadic fever until the end of the experiment. The fever peak in the two vaccinated challenged groups was concentrated approximately 5 days after the challenge. The number of febrile animals from group C was more than group A, with sporadic fever continuing until the end of the experiment.

**Figure 4:**
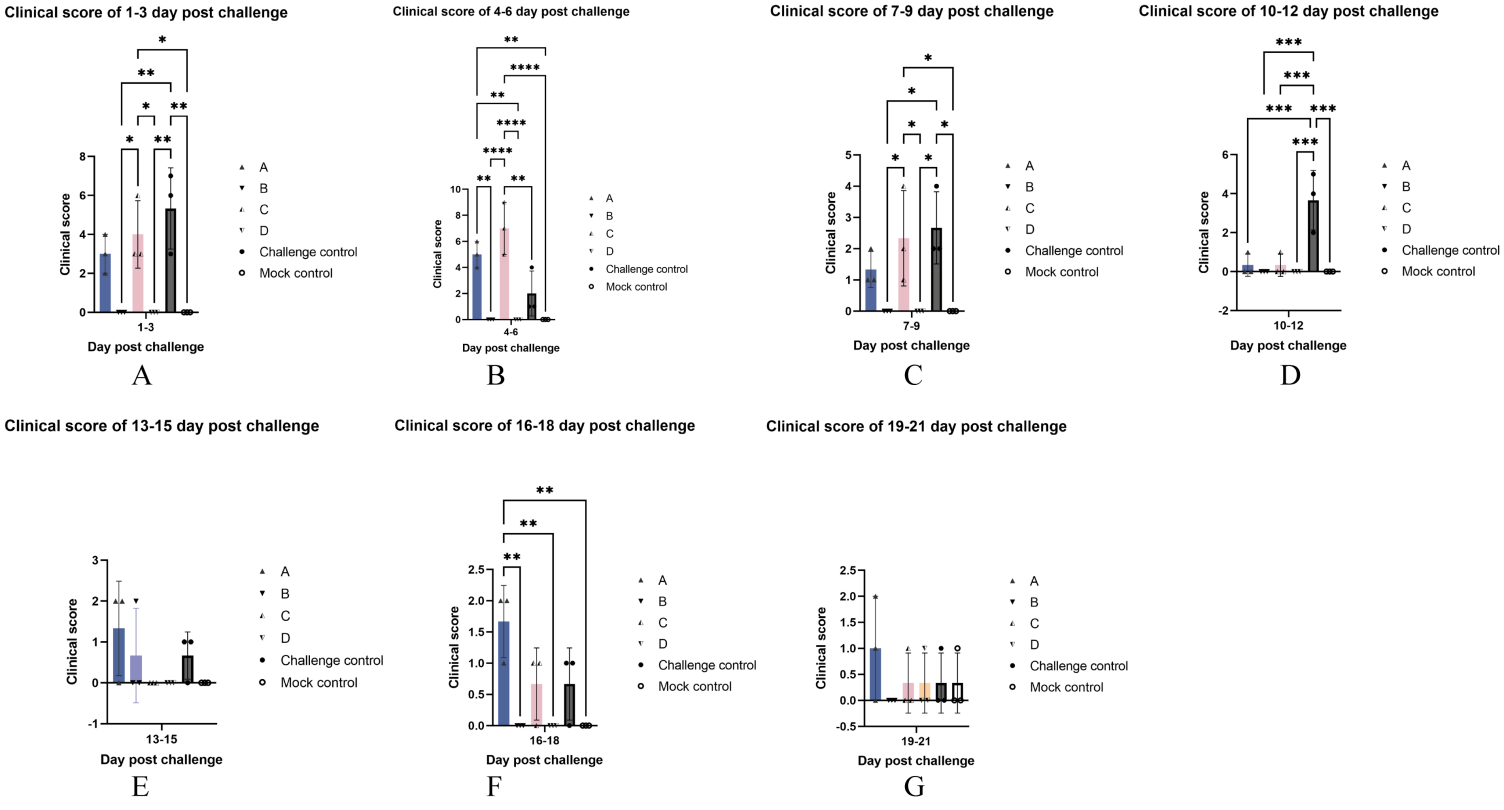
Clinical scores. The data are expressed as the mean ± S.D. of the number of pigs alive at the time of the sample collection. The clinical scores of each group were compared every three days after the challenge: 1–3 **(A)**, 4–6 **(B)**, 7–9 **(C)**, 10–12 **(D)**, 13–15 **(E)**, 16–18 **(F)**, 19–21 **(F)** day post challenge.* indicates a statistically significant difference (*p < 0.05; **p < 0.01; ***p < 0.001; ****p < 0.0001).

As shown in Figure 4A, at 1–3 days after the challenge, all six piglets in the challenge control group had fever, and their average clinical score was the highest, which was significantly higher than that of blank control and two vaccine immune control groups, and had no difference with other challenge groups. The score of group C, which had four piglets with temperatures over 40°C on the second day, was significantly higher than that of the blank control and two vaccine immunization control groups, and there was no difference with other challenge groups. Other than the above, no significant differences were found between the other groups not described.

At 4–6 days after the challenge (Figure 4B), most of the piglets in group C were feverish, and one of them showed signs of lethargy on 6 dpc. The clinical score of group C was significantly higher than that of the control group but had no difference from that of group A. The clinical score of group A was significantly higher than that of blank control and two vaccine immunization control groups, and there was no significant difference between them and challenge control.

Cyanosis was present 7–9 days after the challenge (Figure 4C) in one of the piglets in the challenge control group with the highest clinical score. It was significantly higher than that of blank control and two vaccine immune control groups and had no difference with other challenged groups. Second, the score of group C was significantly higher than the blank control group and the two immune control groups, and there was no difference with group A.

10–12 days after the challenge (Figure 4D), the clinical score of the challenge control group was the highest due to the rectal prolapse of one of its piglets, which was significantly higher than that of all other experimental groups, and there was no significant difference between the other groups.

There was no significant difference in clinical scores among all groups at 13–15 days after the challenge (Figure 4E).

16–18 days after the challenge, the clinical score of group A was the highest (Figure 4F), which was significantly higher than that of blank control and two vaccine immune control groups, and there was no significant difference between the other groups. It is worth noting that a pig from the challenge control group was less active in food and the eating speed was significantly slowed down at 18 dpc.

There was no significant difference in clinical scores among all groups at 19–21 days after the challenge (Figure 4G).

The weight gain of each group of piglets was presented and compared by means of average weight, average daily weight gain, and cumulative average daily weight gain. As shown in Table 4, after adjusting for initial weight, statistical analysis showed that vaccine immunization had a negative effect on productivity growth. The average daily gain in the unvaccinated group was 55 g per day higher than that in the 1 dosage vaccinated group and 27 grams per day higher than that in the 0.1 dosage vaccinated group within 5 weeks after vaccination. Within 5 weeks after vaccination, the 0.1 dosage vaccinated group was 28 g per day higher than the 1 dosage group.

**Table 4:** Average daily weight gain under different immune conditions before the challenge.

	0.1 dosage vaccinated	1 dosage vaccinated	unvaccinated	SEM	*p*-value
**Average weight, kg**					
Week 0	6.13	6.13	6.13		
Week 1	7.62	7.54	7.65	0.12	0.81
Week 2	9.58	9.36	9.66	0.17	0.46
Week 3	12.83ab	12.40b	13.59a	0.30	0.03
Week 4	16.25ab	15.74b	17.34a	0.39	0.03
Week 5	20.91ab	19.91b	21.85a	0.49	0.03
Week 6	24.62	23.88	25.68	0.58	0.09
**Average daily gain in different weeks, g**					
Weeks 0–1	174	162	178	17	0.80
Weeks 1–2	280	261	288	22	0.69
Weeks 2–3	465b	433b	561a	24	0.002
Weeks 3–4	489	478	536	34	0.45
Weeks 4–5	665	595	644	47	0.56
Weeks 5–6	530	568	548	44	0.83
**Cumulative average daily gain, g**					
Week 0–1	174	162	178	17	0.80
Weeks 0–2	227	211	233	12	0.45
Weeks 0–3	306ab	286b	342a	14	0.03
Weeks 0–4	352ab	334b	391a	14	0.03
Weeks 0–5	414ab	386b	441a	14	0.03
Weeks 0–6	434	416	459	14	0.09

According to Table 5, there was no significant difference in cumulative daily gain between the unvaccinated challenged group and the vaccinated group with 1 or 0.1 dosage. Since the feeding environment of the blank control group and the immune control group were changed during the challenge, their weight data and cumulative daily gain data after the challenge were not statistically significant, and the results were for reference only (Supplementary Table S1). However, even with the presence of transition stress in the blank control group, the average body weight was 8.24 kg higher than that of the other groups at 9 weeks (21 dpc) after immunization, i.e., 88 days of age.

**Table 5:** Average daily weight gain under different immune conditions after the challenge.

	0.1 dosage vaccinated challenged group	1 dosage vaccinated challenged group	unvaccinated challenged group	SEM	*p*-value
**Average weight, kg**					
Week 7	29.13	29.38	27.59	1.04	0.45
Week 8	34.77	33.89	31.00	0.97	0.06
Week 9	36.28	36.10	36.77	1.23	0.915
**Average daily gain in different weeks, g**					
Weeks 6–7	727	735	491	75	0.07
Weeks 7–8	807a	644ab	486b	65	0.01
Weeks 8–9	217b	319b	819a	71	0.0002
**Cumulative average daily gain, g**					
Weeks 0–7	464	469	432	21	0.45
Weeks 0–8	507	491	439	17	0.06
Weeks 0–9	474	471	482	20	0.92

### Pathological and histopathological examination

**Figure 5:**
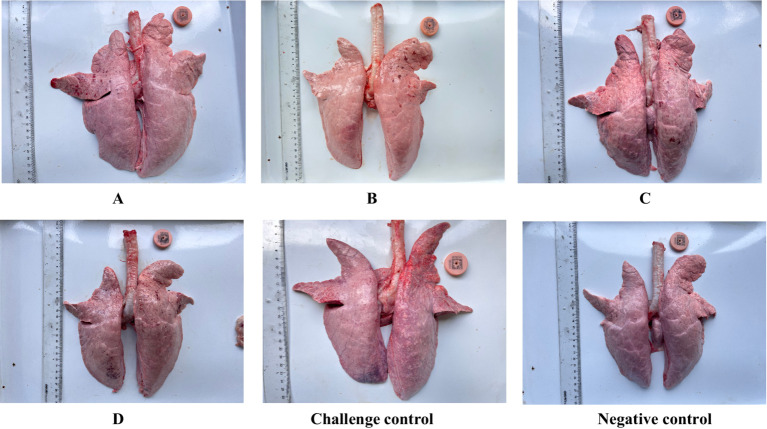
Gross and histological lesions of lungs from different groups of piglets.

**Figure 6:**
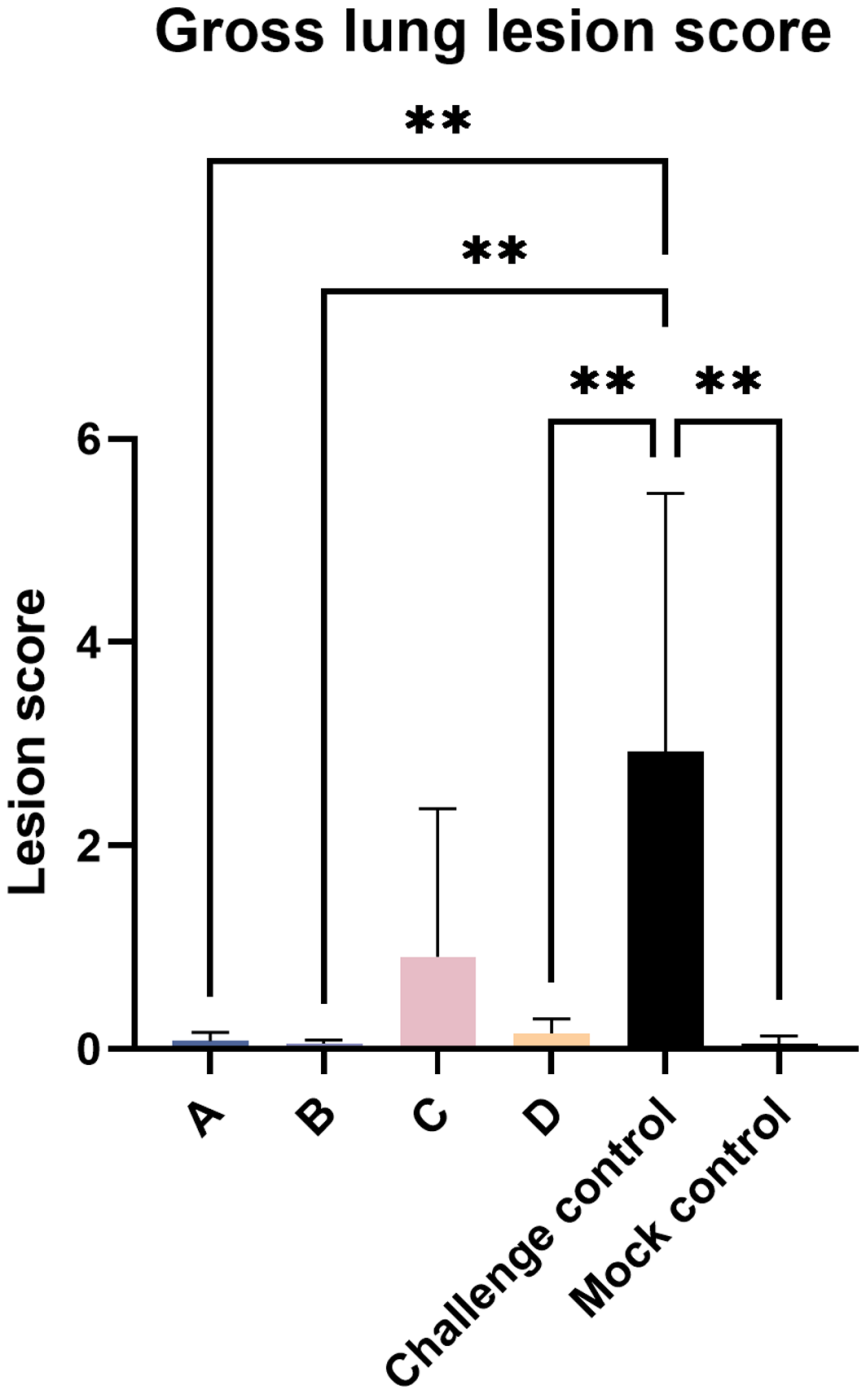
Gross lung lesion score. The data are expressed as the mean + S.D. * indicates a statistically significant difference (**p < 0.01).

Twenty-one days after the challenge, all piglets were euthanized. At necropsy, the pathological changes in the Lungs were observed as shown in Figure 5. The pulmonary carnification of lungs from the challenge control group was more serious and the highest overall score was 6.06, compared to 3.5 in group C and 0.11 in group A. The degree of lung lesions was also scored. The challenge control group had the highest score and the most severe lesions, which was significantly higher than the other experimental groups except group C, and there was no significant difference between the other groups (Figure 6).

**Figure 7:**
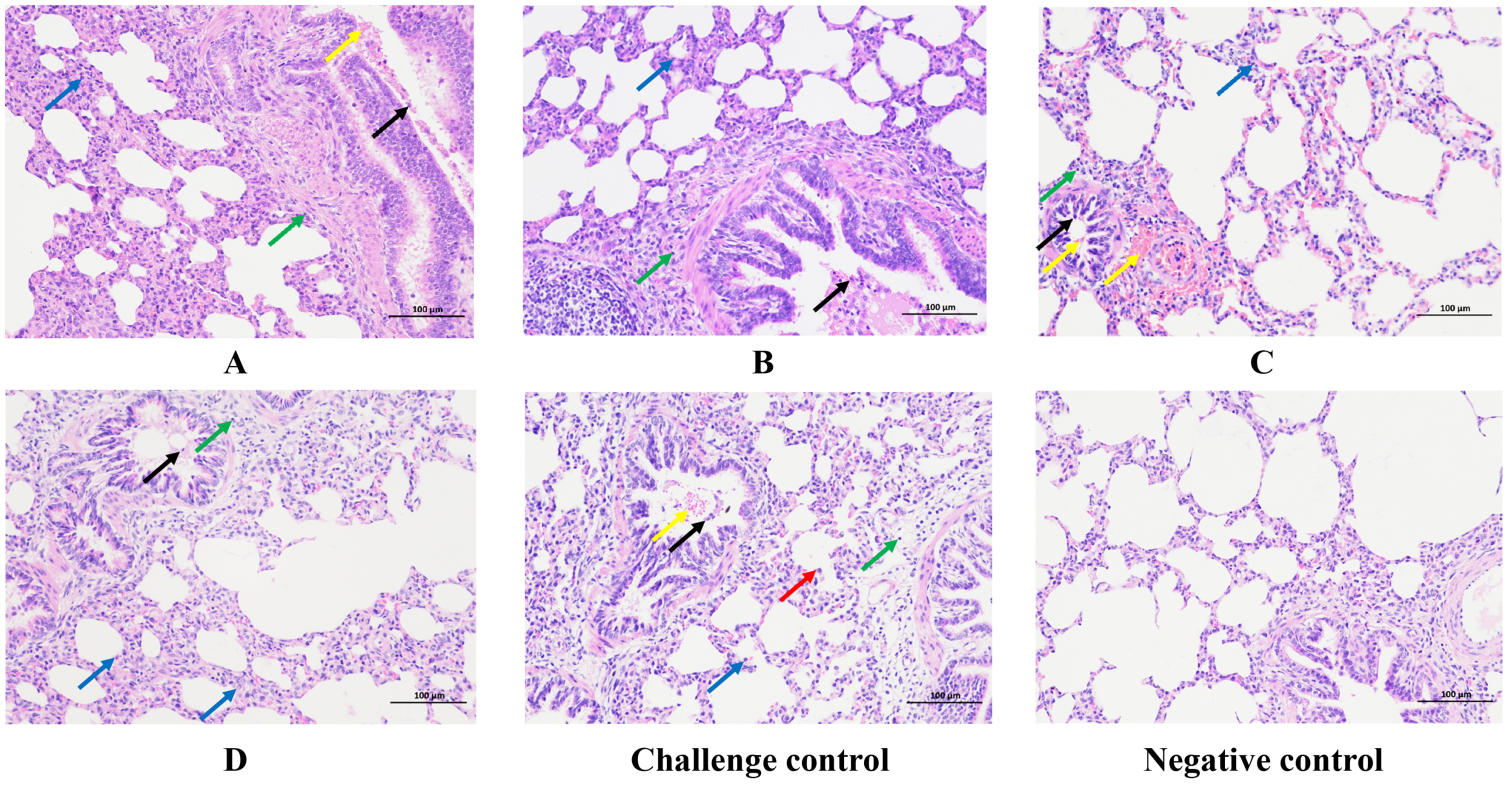
Typical HE manifestations of the lungs from each group. Blue arrows indicate granulocyte infiltration. Green arrows indicate lymphocyte infiltration. Black arrows indicate shed epithelial cells. Yellow arrows indicate hemorrhage. Red arrows indicate macrophage infiltration. Original magnification, 200 × .

Histopathological microscope observation showed that the lungs of the challenged control group showed a small amount of granulocyte infiltration, widened alveolar septum, narrowed alveolar cavity, a large amount of alveolar expansion, and macrophage infiltration was rare in the cavity. A small amount of lymphocyte infiltration is seen around the blood vessels, and bronchus, local bronchial hemorrhage, and exfoliated epithelial cells exist in the bronchial lumen (Figure 7). All immunized groups showed infiltration of alveolar wall granulocytes, a small amount of lymphocyte infiltration around blood vessels and bronchi, and the presence of shed epithelial cells in the bronchial lumen, while bronchial bleeding only existed in group A and group B of all the immunized groups.

**Figure 8:**
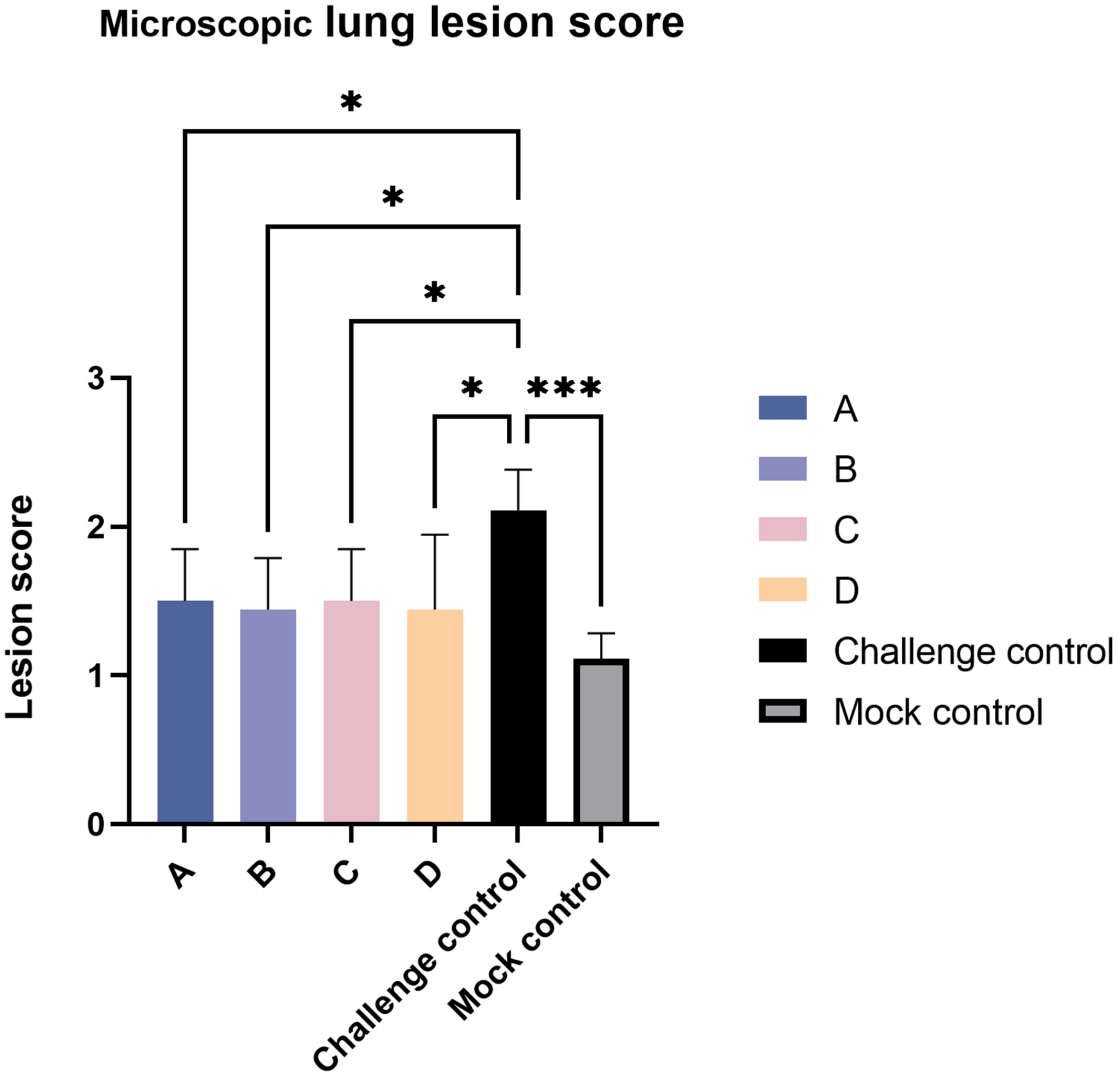
Lung tissue section score. The data are expressed as the mean + S.D. * indicates a statistically significant difference (*p < 0.05; ***p < 0.001).

Histopathologic sections were scored according to the preceding Table 3. The challenged control group had the majority of serious lesions, and its score was significantly higher than that of the other test groups; the score of the blank control group was the lowest, and there was no significant difference between the blank control group and other experimental groups except that the score was significantly lower than the challenge control group (Figure 8).

**Figure 9:**
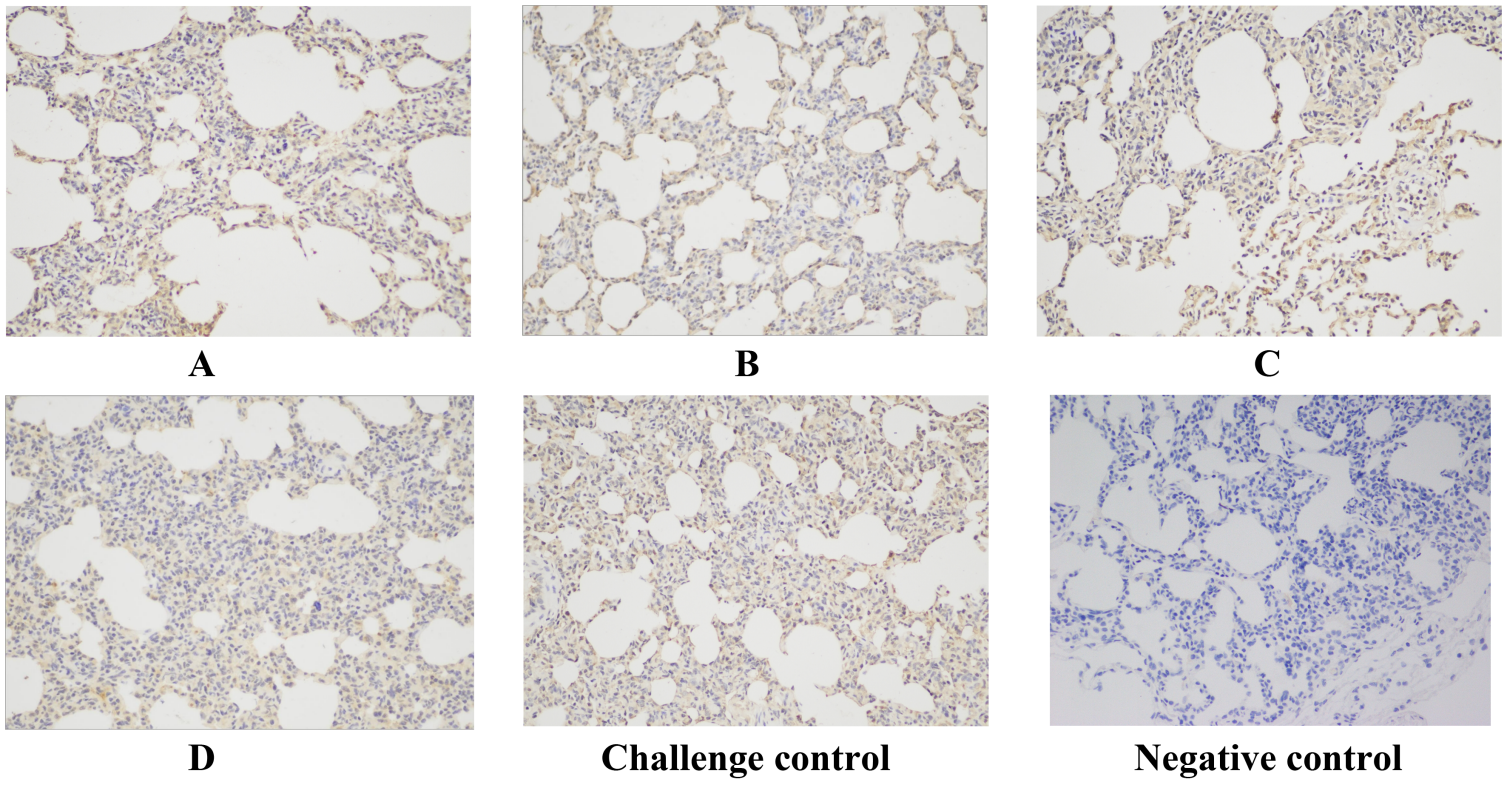
Typical IHC manifestations. Original magnification, 200 × .

**Figure 10:**
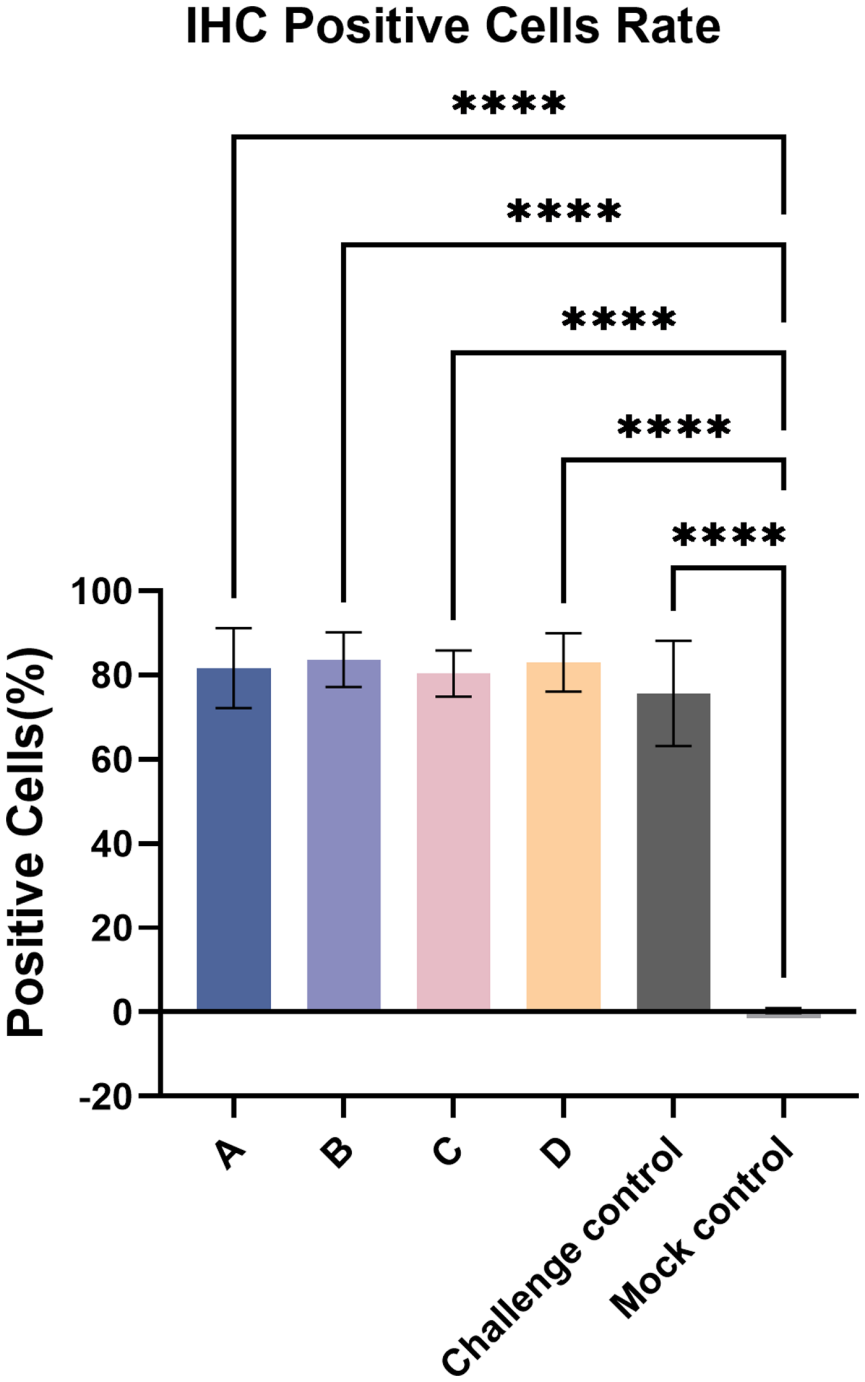
IHC-positive cells rate. The data are expressed as the mean ± S.D. * indicates a statistically significant difference (****p < 0.0001).

Immunohistochemistry (IHC) staining of the lung was also performed to detect the viral antigen, and typical IHC manifestations are shown in Figure 9. Positive cells were counted, and the positive cell rate was calculated (Figure 10). The positive cell rate of the blank control group was the lowest and significantly lower than that of the other experimental groups, and there was no significant difference between the other groups.

### Viremia examination

**Figure 11:**
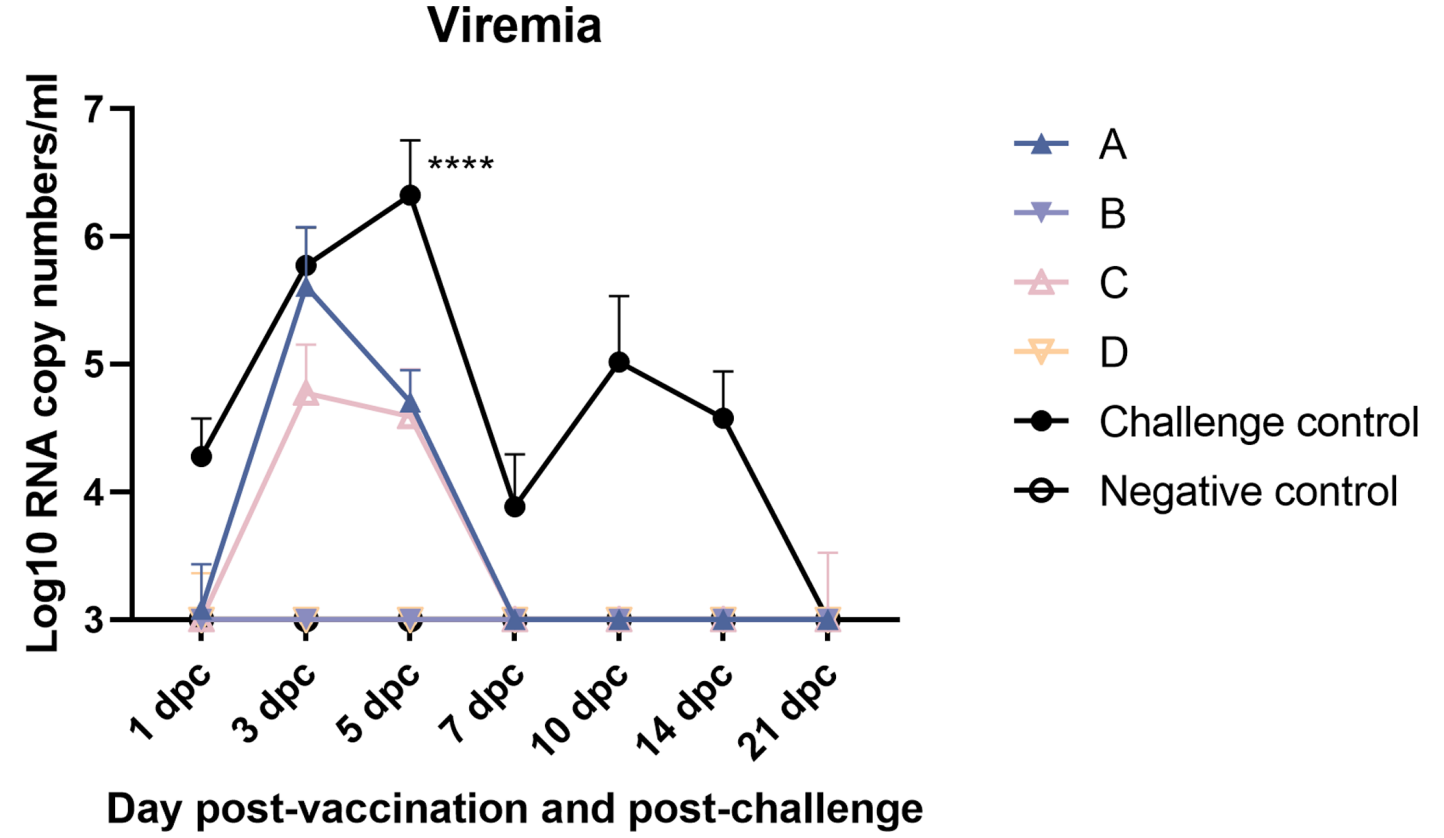
Viremia after NADC34-like DX PRRSV challenge (* indicates a statistically significant difference) (****p < 0.0001).

Shortening the duration of viremia is a key index to evaluate the efficacy of vaccine immunity (Figure 11). Pig serum samples were taken 1, 3, 5, 7, 10, 14, and 21 dpc for viremia assessment. At 5 dpc, the virus load in the serum of the challenge control group was highest and significantly higher than that of the other experimental groups. There was no significant difference between the other groups. The effect of shortening the detoxification period in group C was similar to that in group A (The duration of viremia was slightly longer than or equal to 5 days).

### Serological test

**Figure 12:**
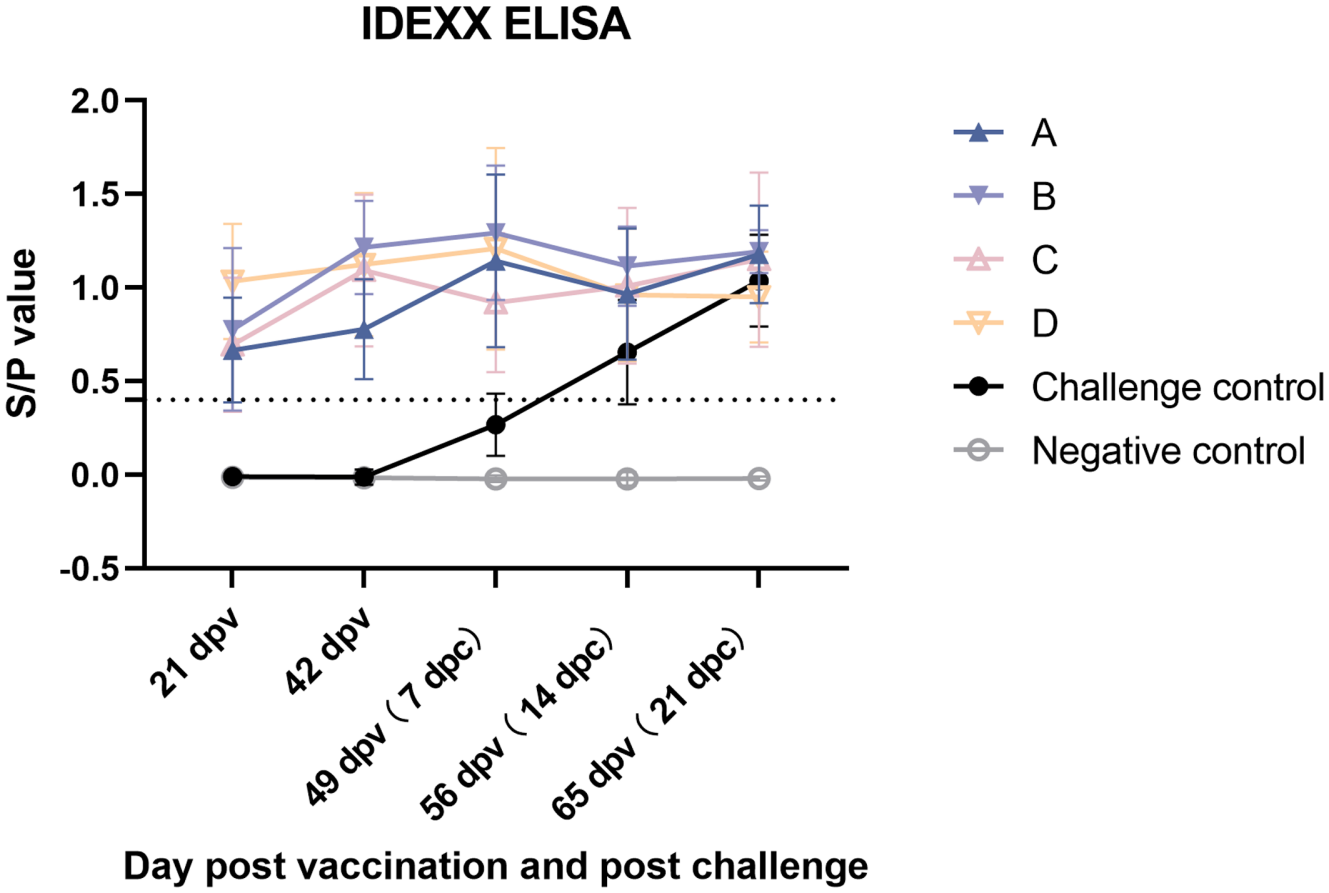
PRRSV-specific antibodies in each group following a PRRSV vaccination or challenge. The threshold for seroconversion was set at a sample-to-positive (s/p) ratio of 0.4 complying with the manufacturer’s guidelines. Each bar represents the average for five piglets ± SEM.

The changes in PRRSV-specific antibodies were monitored from 21 dpv and assessed using an IDEXX ELISA kit. All vaccinated groups in 21 dpv turned positive and maintained an upward trend at 42 dpv (0 dpc) (Figure 12). At 7 dpc, the antibody level of group C decreased, and the average level was lower than that of other vaccine groups and then increased. By the end of the experiment, group B had the highest level of antibodies, followed by group A, group C, challenge control group, and group D.

## Discussion

The pig industry has suffered greatly as a result of NADC34-like PRRSV since it was first identified in the United States (5). Numerous publications from China have demonstrated the variable pathogenicity of PRRSV that resembles NADC34 (27–30). NADC34-like PRRSV DX strain, the strain used in this experiment, is the most stable and virulent of the isolated strains. In the challenge control group of this experiment, the typical clinical symptoms of PRRSV (transient fever, loss of appetite, etc.), and histopathological changes caused by it can also be seen.

The IA/2014/NADC34 prototype was proved highly pathogenic to piglets. While having a lower pathogenicity than the IA/2014/NADC34 strain, the DX strain can cause a longer duration of viremia and produce a larger serum viral load. It may have been reaffirmed that there is no positive correlation between viremia and the pathogenicity of NADC34-like PRRSV strains, and the use of viremia to determine the pathogenicity of NADC34-like PRRSV strains does not seem to be applicable (11).

In 2005, the vaccine against PRRSV was first released in China. For almost 20 years, China has used vaccines to prevent and control PRRSV. However, the current vaccines are not fully effective due to the repeated outbreaks of PRRS and the emergence of new PRRSV variants (9, 31), so the current clinical hope is to reduce the loss by reducing symptoms through immunization. Although safe, inactivated PRRSV vaccines are not effective against wild-type infections because they do not cause cell-mediated immunity (CMI) responses or the generation of particular PRRSV antibodies (32–34). The findings of a study showed that MLV offers significant cross-protection against the NADC30-like virus (35). It has come to our knowledge that Ingelvac PRRS MLV has been evaluated for protection against NADC34-like PRRSV (26). To date, several efficacy studies have been conducted on commercial PRRSV vaccines against different types of PRRSV (30, 31). In our research in the past, we have found that reduced dosages of Ingelvac PRRS MLV can provide better protection for NADC30-like PRRSV challenge. As a consequence, we tried different dosages to evaluate their protective effects against prevalent strains of the NADC34-like virus.

After the challenge, when comparing clinical scores, we found that the MLV vaccine immunization could relieve symptoms (Figure 4). As for gross lung lesion score and microscopic lung lesion score (Figures 6, 8), the score of the vaccine-immunized group was significantly lower than that of the challenge control group, and the performance of the reduced dosage group was better than that of the original dosage group. MLV vaccine immunization also shortened the detoxification period, and the effects of the two groups were similar (Figure 11). In our study, the Ingelvac PRRS MLV (VR-2332) vaccination still had certain toxic side effects and may be dose-dependent, which were reflected in increased body temperature, decreased daily gain, and aggravated lung lesions after immunization.

The majority of these studies talk about vaccine protection from a pathological point of view, so we also looked at the actual impact of vaccines on production. There was no significant difference in daily weight gain in the challenged groups (Table 5), that is, vaccine immunization could not improve the yield loss caused by the virus.

By using different assays, we verified again that the MLV vaccine can provide piglets with some protection against NADC34-like PRRSV. However, the 0.1 dosage Ingelvac PRRS MLV vaccination showed greater benefits in our study. Therefore, taking into account the cost, side effects, and subsequent protective effects, we can adjust the immune dosage appropriately after further investigation to ensure safety, improve production efficiency, and reduce immunization costs.

## Data Availability

The datasets presented in this study can be found in online repositories. The names of the repository/repositories and accession number(s) can be found in the article/supplementary material.
